# Antimicrobial Properties of a Copper/Silicone Composite Membrane Prepared Using a Two-Step Immersion Process in Iodine and Copper Sulfate Solutions

**DOI:** 10.3390/membranes12111049

**Published:** 2022-10-27

**Authors:** Junpei Takeshita, Shiho Aoki, Risei Wada, Ayako Osawa, Jun Sawai

**Affiliations:** 1 Department of Nutrition and Life Science, Faculty of Health and Medical Sciences, Kanagawa Institute of Technology, 1030 Shimo-Ogino, Atsugi 243-0292, Kanagawa, Japan; 2 Faculty of Applied Bioscience, Kanagawa Institute of Technology, 1030 Shimo-Ogino, Atsugi 243-0292, Kanagawa, Japan

**Keywords:** polydimethylsiloxane, antibacterial activity, biofilm, copper, iodine, nanoparticle, silicone membrane, immersion process

## Abstract

Silicone (polydimethylsiloxane) materials are widely used in various applications. Due to microbe adherence and biofilm formation at the surface of silicone materials, silicone materials must possess antibacterial properties. To achieve this, we prepared copper (Cu)–silicone composite membranes using a simple two-step process of immersion in iodine and copper sulfate solutions. Subsequent scanning electron microscopy revealed Cu nanoparticles (CuNPs) of 10 to 200 nanometers in diameter on the silicone membrane surface, which were identified as copper iodide using energy-dispersive X-ray spectroscopy. The mechanical strength of the material did not change significantly as a result of the two-step immersion treatment and the Cu/silicone membrane showed excellent antibacterial efficacy against *Escherichia coli* and *Staphylococcus aureus*, maintaining *R* > 2 even after a physical impact such as stomacher treatment. Additionally, the Cu ions eluted from the Cu/silicone membrane remained at very low concentrations, suggesting firm immobilization of CuNPs on the silicone membrane. This proposed antimicrobial treatment method does not require special equipment, can be performed at room temperature, and has the potential for use on silicone materials other than membranes.

## 1. Introduction

Polydimethylsiloxane-based silicone materials are widely used in various products, such as packing materials, cooking utensils, and medical equipment [1,2,3,4,5,6]. In food processing and clinical use, silicone materials (e.g., tubes, packing materials, and membranes) often come into contact with water, which promotes the adhesion of microorganisms and biofilm formation, thereby increasing the possibility of food poisoning and infections, degradation of the material, and manufacturing defects. Therefore, preventing microbial adhesion and biofilm formation on silicone materials is of great importance [7].

Several methods have been developed for incorporating antimicrobial properties into silicone materials. Although they effectively produce antimicrobial-finished silicone materials, these methods require high amounts of energy, long reaction times, and/or a series of complicated preparation steps, such as immobilization of hydrogen-peroxide-producing enzymes (e.g., cellobiose dehydrogenase) via ultrasound-assisted coating [8], immobilization of short peptides (tryptophan-, arginine-, and lysine-rich) through surface grafting polymerization induced by plasma ultraviolet exposure [9], direct grafting of poly(N-vinylimidazole) on silicone by gamma-ray irradiation [10], coating Ag-doped hydroxyapatite onto PDMS using thermal evaporation (0.01 Pa and >1100 °C) [11], and coating with copper (Cu) ions grafted chitosan using the pneumatic spraying method [5]. Graphene oxide nanocomposites and chitosan–carbon nanotube materials produced by ultrasound-assisted synthesis are also expected to be applied to the antimicrobial surface treatment of silicone [12,13].

In our previous study [14], we prepared silver (Ag)/silicone composite membranes using a simple two-step process of immersion in iodine and silver nitrate solutions. The surface of the prepared Ag/silicone membrane was dotted with Ag nanoparticles (NPs) ranging in size from several nanometers to several tens of nanometers and exhibiting very high antibacterial properties. These Ag/silicone membranes were also highly durable when subjected to acid treatment and physical stress, exhibiting a low elution of Ag^+^. Importantly, this simple antimicrobial treatment of silicone membranes using a two-step immersion process did not require any special equipment and was performed at 25 °C (room temperature).

Silver and copper are the metals most widely used for their antimicrobial properties. Kawakami et al. [15] evaluated the antibacterial activity of 21 metal elements against *Staphylococcus aureus* and *Escherichia coli* and ranked Ag and Cu as first and second, respectively. Copper is known to be effective in reducing the growth of various microorganisms [16,17,18]. The antimicrobial efficacy of technologically appealing materials containing copper-based active powders or pigments has been reported in fabrics, paints, and coatings [19], aqueous copper solutions [20], complex copper species [21], copper-alloyed stainless steel [22], and CuNPs-embedded hydrogel for wound healing [23]. In the present study, we prepared Cu/silicone composite membranes using a two-step immersion process involving Cu and characterized the membrane properties and antimicrobial activity of prepared membranes. The same method applied to silver could also be applied to copper, which also had high antimicrobial activity. The results also provide a choice between silver and copper depending on the characteristics of the antimicrobial target.

## 2. Materials and Methods

### 2.1. Preparation of Cu/Silicone Composite Membranes

We prepared the Cu/silicone membrane following our previously described method [14]. The silicone membrane (0.3 mm-thick; ASONE, Osaka, Japan) was cut into 50 × 50 mm pieces. 

An I_2_-KI solution was prepared by dissolving iodine in a 3.3 M potassium iodide solution to a concentration of 0.15 M, with the concentration of iodine almost reaching the saturation concentration. Both reagents were purchased from FUJIFILM Wako Pure Chemical Co. (Osaka, Japan). The silicone membrane was then immersed in the I_2_-KI solution at 25 ± 2 °C for 24 h. The optimal iodine concentration and immersion time were determined in the previous study [14]. The amount of iodine contained in the silicone film was the most significant factor affecting its antimicrobial activity; however, the antimicrobial efficacy did not increase after immersion for more than 24 h.

Subsequently, the membrane was removed from the I_2_-KI solution, the residual solution was wiped off its surface, and the membrane was immersed in a 0.1, 0.3, or 0.5 M copper sulfate (CuSO_4_) (FUJIFILM Wako Pure Chemical) solution at 25 ± 2 °C for 24 h. Afterwards, the surface of the membrane was washed with pure sterile water and wiped with 70% ethanol before use. Preliminary experiments to determine the concentration of CuSO_4_ solution showed that Cu/silicone membranes prepared with 0.1 M CuSO_4_ had an antimicrobial activity value (*R*; calculated as shown in Equation (1)) of no more than 2. In contrast, membranes prepared with 0.3 M and 0.5 M CuSO_4_ solutions showed almost equal antimicrobial activity with *R* > 2. Notably, the membrane prepared with 0.5 M CuSO_4_ solution showed a more stable *R* value. Therefore, subsequent membranes were prepared using a 0.5 M CuSO_4_ solution.

The weight of the silicone membrane was measured before and after the immersion treatment, and the amount of Cu bound to the silicone membrane was calculated based on the recorded weight difference.

### 2.2. Scanning Electron Microscopy 

The surface of the Cu/silicone membrane was characterized using scanning electron microscopy (SEM) (Model SU 9000, Hitachi High-Technologies Co., Tokyo, Japan). The unprocessed silicone membrane was also characterized by SEM (JSF-7001F, Joel Ltd., Tokyo, Japan).

Analysis of the elemental particles on the silicone membrane was performed using SEM–energy-dispersive X-ray spectroscopy (EDS). SEM observation and EDS analysis were conducted using a JSM-IT800 (JASCO Co., Tokyo, Japan) and an Octane Elect EDS system (AMETEK Co., Ltd., Berwyn, PA, USA), respectively.

### 2.3. Tensile Strength Test

The mechanical strength of the silicone membrane after the two-step immersion process was also investigated. According to the specifications of the Japanese Industrial Standard (JIS) K6251-6 [24], the Cu/silicone membrane was cut into a dumbbell shape with a parallel section width of 3.7 mm and a length of 25 mm using a cutter and then uniaxially stretched at a tension rate of 10 mm/min, which was determined in a previous study [25]. Young’s modulus (*E*) was estimated from the initial gradient of the obtained stress–strain curves, and the mechanical strength of the Cu/silicone membrane was thereby evaluated. An unprocessed silicone membrane was measured as the control.

### 2.4. Elution Test

The prepared Cu/silicone membrane was immersed in 100 mL of distilled water in a 100 mL beaker and then agitated using a magnetic stirrer. Samples were removed from the beaker at regular intervals and used to determine the concentration of released Cu ions using a Copper Assay Test Kit (Metallogenics Co., Ltd., Chiba, Japan). A reagent was added to a portion of the sample according to the kit manufacturer’s instructions and the optical density was measured at 580 nm using a UV/VIS spectrometer (UV-2075; JASCO, Tokyo, Japan).

### 2.5. Antibacterial Efficacy Test

*E. coli* NBRC 3301 and *S. aureus* NBRC13276 obtained from the National Institute of Technology Evaluation Biological Resource Center (NBRC, Kazusa, Japan) were used to test the antibacterial efficacy of the Cu/silicone membrane. Both strains were incubated in nutrient broth (NB: Eiken Chemicals, Tokyo, Japan) with shaking (110 strokes/min) for 20 h at 37 °C. The antibacterial efficacy (*R*) of the Cu/silicone membrane was evaluated according to JIS Z 2801 [26]. Cultures were then washed and resuspended in 1/500 NB at approximately 10^6^ CFU/mL. The bacterial suspension was stored in an ice water bath before use. A polyethylene film with a thickness of 0.05 mm (ASONE) was cut into 40 × 40 mm pieces and wiped with 70% ethanol for disinfection. Then, 0.1 mL of bacterial suspension was inoculated on the Cu/silicone membrane, covered with the polyethylene film, and incubated at 35 °C and a relative humidity of at least 90% for 24 h. The Cu/silicone membrane was placed into a sterilized stomacher filter bag (bioMérieux Japan, Tokyo, Japan) containing 10 mL of soybean casein digest broth with lecithin and polysorbate 80 (SCDLP; Eiken Chemicals). The bag was treated for 1 min with the stomacher (bioMérieux Japan) to wash the bacterial cells out of the membrane. The solution in the stomacher filter bag was diluted with sterile saline (0.85%), inoculated onto nutrient agar (Eiken Chemicals), and incubated at 35 °C for 24 h. Duplicate agar plates were used for each dilution. The number of colonies formed was determined for evaluation of the viable bacterial counts on the Cu/silicone membrane. An untreated silicone membrane was used as a control. Three pieces of Cu/silicone membrane and three pieces of untreated silicone membrane were used for each test.

The value of *R* was calculated according to the following equation, with *R* > 2 indicating antibacterial efficacy.

*R* = log (*U*_t_/U_0_) − log (*A*_t_/U_0_) = log (*U*_t_/*A*_t_) (1)

where *U*_t_, *U*_0_, and *A*_t_ represent the following:

*U*_0_: mean logarithm of the number of viable bacteria immediately after inoculation of the unprocessed silicone membrane.
*U*_t_: mean logarithm of the number of viable bacteria after 24 h of the untreated silicone membrane.
*A*_t_: mean logarithm of the number of viable bacteria after 24 h of the Cu/silicone membrane.

### 2.6. Durability Test

The Cu/silicone membrane was placed into the sterilized stomacher filter bag containing 10 mL of sterile saline and underwent stomacher treatment for 1 min. The stomacher-treated membranes were subsequently removed and placed in stomacher bags containing 10 mL of fresh sterile saline for another round of stomaching for 1 min. The same procedure was repeated up to 10 times. The stomacher-treated membrane was repeatedly rinsed with sterile pure water, dried on a clean bench, and evaluated for antibacterial efficacy according to the procedure described above.

### 2.7. Statistical Analysis

All tests were performed in triplicate (*n* = 3). Significant differences between mean values at *p* < 0.05 were determined using BellCurve for Microsoft Excel (Social Survey Research Information Co., Ltd., Tokyo, Japan). 

## 3. Results and Discussion

### 3.1. Cu/Silicone Composite Membrane 

**Figure 1:**
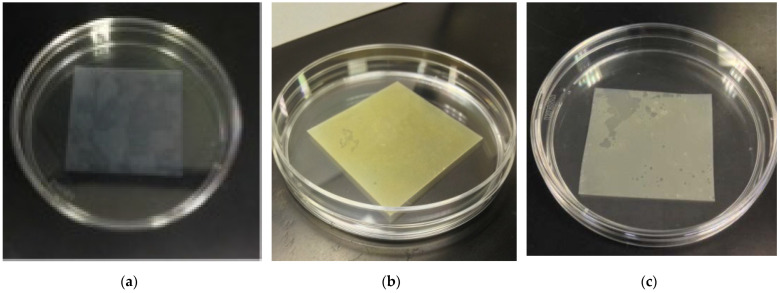
Color change of silicone membrane in the processing stage. (**a**) Unprocessed membrane, (**b**) after KI-I_2_ treatment at 0.15 M I_2_ for 24 h, (**c**) treatment with 0.5 M CuSO_4_ for 24 h after KI-I_2_ treatment.

We observed that the silicone membrane was initially semitransparent (Figure 1a), but turned brown after iodine treatment, indicating the accumulation of iodine in the membrane (Figure 1b). After CuSO_4_ treatment, the membrane became white (Figure 1c), suggesting the formation of copper iodide (CuI).

**Figure 2:**
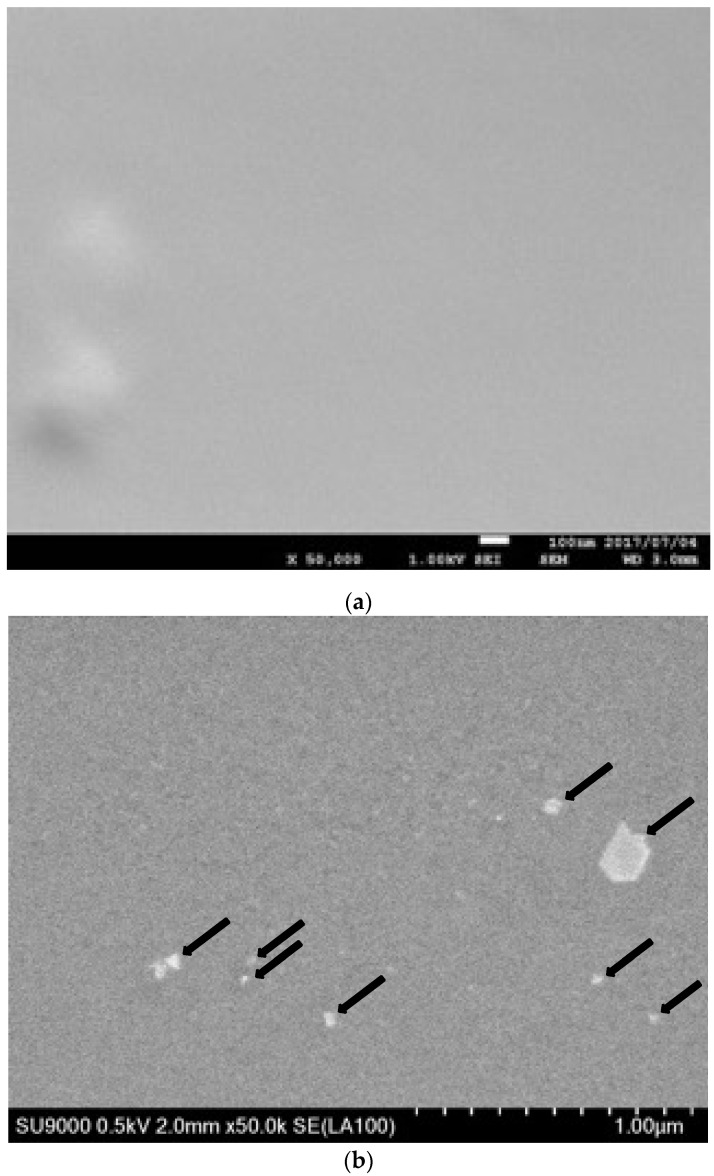
SEM image of (**a**) an untreated silicone membrane and (**b**) a Cu/silicone membrane. Magnification: (**a**) 50,000×, and (b) 500,000×. The Cu/silicone membrane was prepared via a 2-step treatment with KI-I_2_ (0.15 M I_2_, 24 h) and 0.5 M CuSO_4_ (24 h) at 25 ± 2 °C.

The surface of untreated silicone membranes was flat and smooth, as shown in the SEM image (Figure 2a). In contrast, SEM observations revealed that particles 10 to 200 nanometers in diameter were present on the silicone membrane surface as dots after CuSO_4_ treatment (Figure 2b; typical particles are indicated by arrows). We found that the spacing between particles was approximately several hundred nanometers. In addition, we noticed that the particle size on the Cu/silicone membrane was one order of magnitude greater than that on the Ag/silicone membrane [14], and the spacing was one order of magnitude wider. There was also considerable variation in the size of the particles. During the immersion treatment of iodine, there may be local highs and lows in the amount of iodine accumulation in the silicone membrane. However, this difference in particle size is not apparent at the present stage.

**Figure 3:**
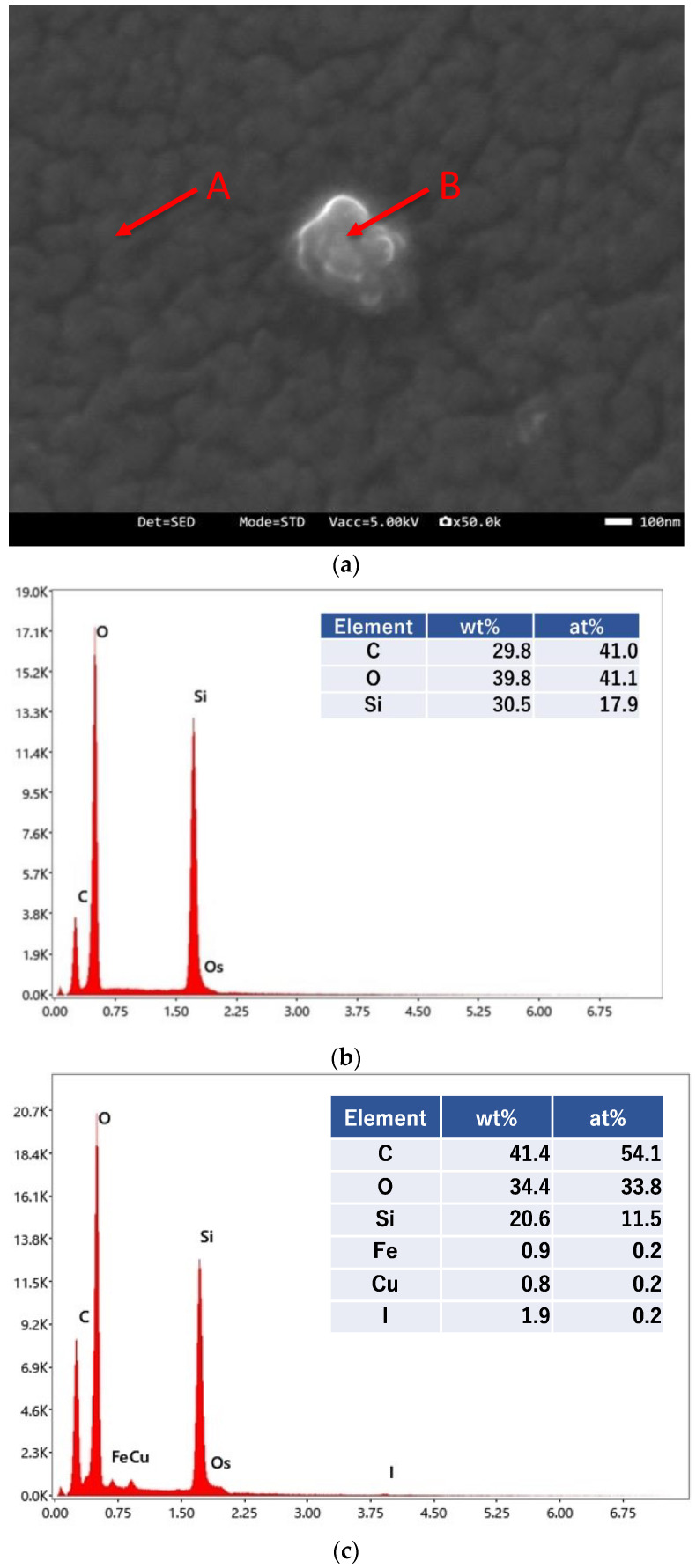
SEM-EDS analyses of the Cu/silicone membrane. (**a**) Bright-field image, (**b**) EDS spectra at the point of arrow A, and (**c**) EDS spectra at the point of arrow B. The Cu/silicone membrane was prepared via a 2-step treatment with KI-I_2_ (0.15 M I_2_, 24 h) and 0.5 M CuSO_4_ (24 h) at 25 ± 2 °C.

The results of SEM-EDS analysis are shown in Figure 3. We detected the presence of Si, C, and O, which are components of the silicone membrane in the area indicated by arrow A in Figure 3a; the peak with Os was due to its use in a deposition for the measurement (Figure 3b). In addition to Si, C, and O, we detected the presence of Cu and iodine (I) in the particle indicated by arrow B (Figure 3c). Considering the weight % and atomic % values of Cu and I, we confirmed that the particle on the silicone membrane was CuI.

The formation of CuI is considered to occur as follows. First, copper ions react with iodide ions incorporated into the silicone membrane to form copper (II) iodide.

Cu^2+^ + 2I^−^ → CuI_2_ (2)

The generated CuI_2_ immediately decomposes into copper(I) iodide and iodine, forming CuI on the silicone membrane [27].

2CuI_2_ → 2CuI + I_2_ (3)

As shown in Figure 2, the size of CuI nanoparticles on the silicone varied considerably. Local analysis could not determine the overall amount of Cu. Thus, we determined the amount of Cu bound to the silicone membrane from the change in the weight of the silicone membrane before and after the immersion treatment. Based on the assumption that Cu was bound to the silicone membrane as CuI, we calculated the amount of bound Cu to be 20.7 ± 7.9 µg/cm^2^. For coating and loading of silicone or other polymeric materials with CuNPs, loadings of 0.5–3% have commonly been reported [17,28], whereas when CuNPs are formed on the surface by chemical reaction, loadings of approximately 0.6–5 µg/cm^2^ have been demonstrated [5,29]. These results indicate that the Cu/silicone membrane obtained by the immersion method has a high loading capacity for coating CuNPs using a chemical reaction.

**Figure 4:**
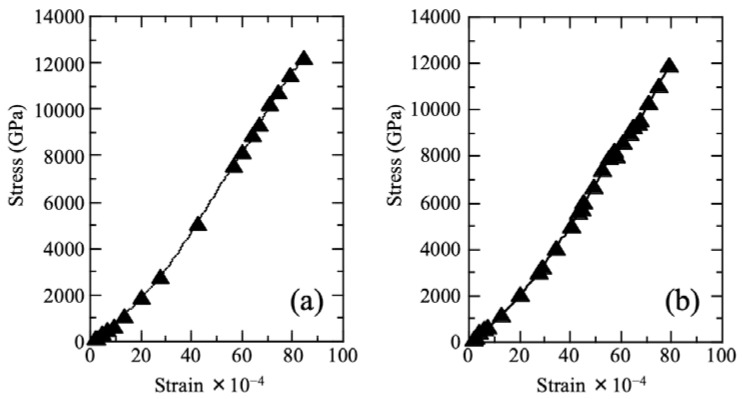
Typical stress–strain curves of unprocessed silicone membrane (**a**) and Cu/silicone membrane (**b**). The Cu/silicone membrane was prepared via a two-step treatment with KI-I_2_ (0.15 M I_2_, 24 h) and 0.5 M CuSO_4_ (24 h) at 25 ± 2 °C.

The typical stress–strain curves of Cu/silicone membranes and untreated membranes are shown in Figure 4. We did not detect any differences between the stress–strain curves of the untreated and treated membranes. The Young’s modulus of the untreated membrane was 7.9 ± 1.2 MPa, which is consistent with the typical values reported for silicone membranes (3–14 MPa) [30]. We observed that the Cu/silicone membrane had a slightly higher Young’s modulus of 8.9 ± 1.1 MPa, which, however, did not differ significantly from that of the untreated membrane (*p* > 0.05). Based on this, we concluded that the mechanical strength of the silicone membrane did not change significantly following the two-step treatment process.

### 3.2. Cu Elution from the Cu/Silicone Membrane

**Figure 5:**
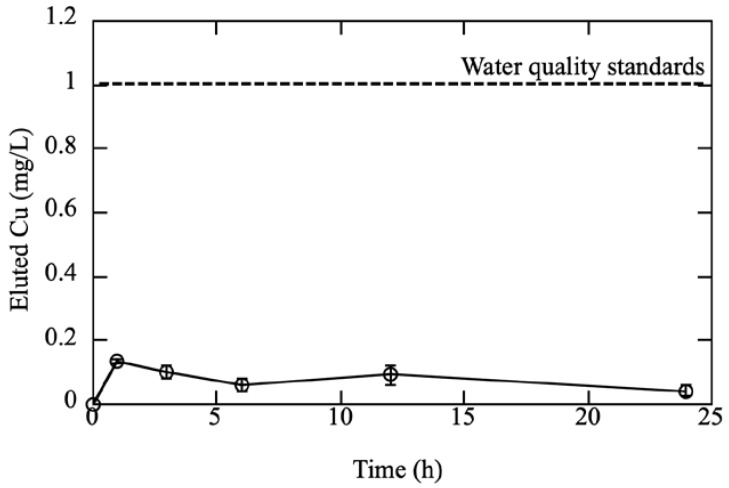
Elution of Cu ions from the Cu/silicone membrane. Data points with bars represent the mean ± standard error (*n* = 3). The Cu/silicone membrane was prepared via a 2-step treatment with KI-I_2_ (0.15 M I_2_, 24 h) and 0.5 M-CuSO_4_ (24 h) at 25 ± 2 °C.

We also investigated the elution of Cu ions from the Cu/silicone membrane. In Figure 5, data points and error bars represent the mean ± standard error of the mean. Based on the assay results, we found that the Cu concentration increased initially but stabilized after 1 h. We determined that after 1 h, approximately 0.1 mg/L of Cu ions was eluted from the Cu/silicone membrane. We hypothesize that the Cu did not solidly crystallize on the Cu/silicone membrane during the preparation process and was therefore eluted in the form of ions at a low concentration. We further observed that the concentration of eluted Cu ions was ~0.1 mg/L after 24 h. The current Water Quality Standards for Water Supply from the Ministry of Health, Labor and Welfare in Japan [31], stipulate that the standard value for Cu should be ≤1 ppm. In the present study, we found that the concentration of Cu ions was <1 ppm for all elution durations. Given the solubility of CuI at 25 °C (0.42 mg/L), we assumed that the concentration of CuI would not increase to >0.42 ppm with further extension of the elution time. Together with the results of the stomacher treatment (Table 1 and Figure 5), these findings indicate that Cu was firmly attached to the silicone membrane and did not detach easily. Although the safety of this composite membrane requires further study, it has the potential to be a relatively safe material at this stage.

### 3.3. Antibacterial Efficacy and Its Durability

The antimicrobial efficacy of the prepared Cu/silicone membrane according to JIS Z 2801 [26] is shown in Table 1. We calculated the *R* value using Equation (1), with *R* > 2 indicating antibacterial efficacy. We observed that, although the *R* values of the Cu/silicone membrane were lower than those of the Ag/silicone membrane [14], they had an *R* value > 4 when tested against either *E. coli* or *S. aureus*, indicating excellent antibacterial effects. Ag ions are known to have higher antimicrobial activity against bacteria than Cu ions [15], which agrees with our observations of the silicone composite membrane prepared using the described method. Furthermore, even after physical stress treatment through 5–10 repeat stomacher sessions, the *R* value of the Cu/silicone membrane was ≥3 and did not decrease significantly (*p* > 0.05). Thus, we concluded that the Cu/silicone membrane possessed sustainable high antimicrobial activity. As shown in Figure 4, the amount of Cu eluted was small. However, as shown in Table 1, the Cu/silicone membrane exhibited high antimicrobial activity when in contact with bacterial cells or when bacteria adhered to the membrane. If this antimicrobial material is used as a packaging material to target floating bacterial cells, it may not exhibit an appropriate bactericidal effect. Therefore, the antimicrobial silicone material prepared in this study can be regarded as an antimicrobial material that is effective against bacteria that may come into contact with the membrane materials.

There have been few reports on the antimicrobial mechanisms of Cu ions and CuNPs compared with those of Ag and other compounds. Cu ions have been shown to impair membrane function by binding to cell membranes and cell wall components and to lyse bacteria by activating autolytic enzymes [32]. In the cytoplasm, Cu ions can inhibit the function of proteins and enzymes and metabolism by binding to -SH groups and amino acid residues, as well as inhibit RNA and DNA synthesis and cause damage due to reactive oxygen species generated via the Haber–Weiss and Fenton reactions [32]. Raffi et al. [33] and Ruparelia et al. [34] suggested that Cu ions originating from NPs might interact with phosphorus- and sulfur-containing biomolecules, such as DNA and proteins, to distort their structures, thereby disrupting biochemical processes. Chatterjee et al. [35] reported that the action mechanisms of Cu ions and CuNPs are based on the NP-mediated dissipation of cell membrane potential and multiple toxic effects, such as generation of reactive oxygen species, lipid peroxidation, protein oxidation, and DNA degradation in *E. coli* cells. Sharma et al. [36] showed that the CuNP–microorganism interaction induced ROS-generated oxidative stress, resulting in leakage of cytoplasmic components, loss of membrane permeability, and ROS generation, which were the primary causes of CuNP-induced bacterial cell death. At this time, it is difficult to precisely determine the mechanisms by which copper/silicone membranes exhibit antimicrobial activity. However, we speculate that the interaction between the bacterial cells adhering to the CuI-coated surface and CuI nanoparticles causes CuI to decompose into Cu and iodine (e.g., 2CuI → 2Cu + I_2_ or 2CuI → Cu + CuI_2_). The Cu produced here may become Cu ions. This copper ion and iodine act and exert antimicrobial activity, resulting in increased cell membrane permeability and oxidation or leakage of cell contents. In applying the Cu/silicone membrane prepared by the two-step immersion treatment, including the generation of reactive oxygen species, further study is necessary to examine the antimicrobial membrane effect.

## 4. Conclusions

The proposed method in this study is straightforward, requiring only a two-step immersion process without involving the use of any special equipment. The Cu/silicone membrane produced by this method showed high antimicrobial activity and maintained its mechanical strength, while Cu elution was suppressed to a very low level. The method for antimicrobial treatment of silicone materials described in this study could be used not only for the generation of thin films but also for the processing of materials after molding. With reports of outbreaks of various infectious diseases, the number of pathogenic microorganisms in our everyday environment needs to be reduced. Silicone is now widely used in various products, such as gaskets, cooking utensils, medical materials, and separation membranes, and, therefore, the proposed antimicrobial treatment has the potential for application in a wide range of commonly used items that would benefit from antimicrobial properties.

## Figures and Tables

**Table 1:** Antibacterial efficacy of Cu/silicone membranes and their durability after stomacher treatment.

Membrane	The Number of Stomacher Treatment	*R* Value	
		*E. coli*	*S. aureus*
Cu/silicone	0	4.2 ± 0.5	4.5 ± 0.7
	5	2.9 ± 0.5	3.1 ± 0.1
	10	3.1 ± 0.1	3.5 ± 0.1
Ag/silicone *	0	>6.0	>6.0
	5	-	-
	10	>6.0	>6.0

* Aoki et al. (2018) [14]. - Not tested.

## Data Availability

The data presented in this study are available on request from the corresponding author.

## Abbreviations

CFU	Colony-forming unit
EDS	Energy-dispersive X-ray spectroscopy
*E*	Young’s modulus
*E. coli*	*Escherichia coli*
JIS	Japanese Industrial Standard
NB	Nutrient broth
NP	Nanoparticle
SCDLP	Soybean–casein digest broth with lecithin and polysorbate 80
SEM	Scanning electron microscope
*S. aureus*	*Staphylococcus aureus*
